# Prediction of Fusion Hole Perforation Based on Arc Characteristics of Front Image in Backing Welding

**DOI:** 10.3390/ma13214706

**Published:** 2020-10-22

**Authors:** Yu Cao, Xiaofei Wang, Xu Yan, Chuanbao Jia, Jinqiang Gao

**Affiliations:** 1 MOE Key Lab for Liquid–Solid Structure Evolution and Materials Processing, Shandong University, Jinan 250061, China; caoyu1506@163.com (Y.C.); welding09wang@163.com (X.W.); hangangyanzi@163.com (X.Y.); jiachuanbao@sdu.edu.cn (C.J.); 2 Institute of Materials Joining, Shandong University, Jinan 250061, China

**Keywords:** one-side welding with back-formation, fusion hole, visual sensing, prediction, arc characteristics

## Abstract

In one-side welding with back-formation, the weld is penetrated after the fusion hole is perforated. Therefore, judging whether the fusion hole is perforated is very important to realize autocontrol of penetration in one-side welding with back-formation process. Previous researches mainly focused on the morphological characteristics of the molten pool and fusion hole to judge the weld penetration state. Sometimes it is difficult to obtain the morphological characteristics of the molten pool, keyhole and fusion hole and image processing is complex. In this paper, a visual detection system of fusion holes based on visual sensing is constructed to obtain the real-time fusion hole images in backing welding. It is found that the arc characteristics in the front images contain abundant information about the perforation of fusion hole. An image processing program is developed based on MATLAB software, and the arc characteristic parameters in front images are obtained. Taking the arc characteristic parameters as the input, obtaining the penalty function and the kernel function parameters through the cross validation and grid search method, a prediction model of fusion hole perforation based on the support vector machine is put forward. The accuracy for prediction samples is 88%. By analyzing the misidentified samples, it is found that some of the newly perforated images are predicted as nonperforated ones, which has little influence on the penetration control of the weld.

## 1. Introduction

The technology of one-side welding with back-formation is mainly applied to welding structures with limited space in the back. In order to ensure penetration, the technology generally requires a certain reserved gap for butt welding [1,2,3]. In the pressure vessel and pipeline industry, the technology of one-side welding with back-formation is a skill that welders must master. There always exists a fusion hole in the process of butt welding with reserved gap, which is a special phenomenon. The state that the bottom edge of the root under the fusion hole is melted is taken as perforation in this paper. The perforation of the fusion hole ensures the full melting of the root and the penetration of weld. Skilled welders can get the information to judge whether the weld is penetrated by observing the shape and size of the fusion hole in the welding process. Therefore, judging the perforation of the fusion hole in welding is of great significance to realize the welding automation of high-quality one-side welding with back-formation.

Welding is often accompanied by various physical phenomena [4,5], which contain numerous pieces of information closely related to the penetration state of the weld. Many sensing methods are adopted to obtain such information. Some researchers [6,7,8] utilized arc sound, welding voltage and current and other indirect signals obtained in the welding process to detect the welding state. Compared with the nonvisual signal sensing method, the visual signal sensing method has the advantages of high measuring accuracy, large amount of information and noncontact with the work piece, which is widely used in the shape detection of molten pool and keyhole [9,10]. Bae et al. [11] established a set of visual sensing systems for GMAW (gas metal arc welding) and extracted the shape of molten pool from the image of molten pool and surrounding area captured. They calculated the position of wire and molten pool center, realizing a good tracking of weld position with the maximum offset error of 1°, but the weld penetration was controlled according to the measured gap dimension and the visual detection of weld penetration wasn’t realized in the experiment. Bacioiu et al. [12] adopted a neural network to learn the differences between good welds and defective welds and built the relationship between weld quality and morphological information of molten pool captured by HRD (high dynamic range) camera. Zhang et al. [13] obtained the backside images of molten pool and keyhole by using pulse triggered imaging method in K-TIG (keyhole tungsten inert gas) welding, and established the prediction model of the relationship between molten pool and keyhole and weld penetration. Zhang et al. [14,15] built a set of CCD (charge-coupled device) double-sensor visual detection systems. They obtained the size and position information of the keyhole and backside molten pool, realizing the penetration control of stainless steel with the thickness of 11.5 mm. Liu et al. [16,17,18] built a visual detection system for K-TIG welding, which realized the “one pulse, one hole” K-TIG welding process by using a square-wave pulse welding current. Through observing the K-TIG welding process of a stainless steel plate, they got the images of the keyhole in the backside of weld, and extracted the shape and position characteristic parameters of the keyhole. Luo et al. [19] established the relationship between welding parameters and unobservable keyhole geometry through a static neural network and obtained keyhole depth and inclination angle by the dynamic radial basis function neural network observer under varying welding parameters in PAW (plasma arc welding). Bardin et al. [20] built a closed-loop control system for LBW (laser beam welding). Through the focal control system, the laser beam could be kept focused over a wide range of welding parameters and full penetration was realized. With the keyhole image recognition system, the opening degree of the keyhole was analyzed and the energy inputted was adjusted to avoid burn-through and partial-penetration.

For work pieces with complex shapes, it is difficult to sense the weld penetration from the backside of weld, therefore judging the state of penetration based on the visual detection signals from the front is meaningful [21]. The image of the keyhole and molten pool reflects the penetration state of weld. Researchers have built various penetration prediction models based on machine learning [22]. Xia et al. [23] took the front image of the molten pool captured by HRD camera as the input, establishing a classification model of welding state through the CNN (convolutional neural network) method based on the residual network in K-TIG welding. The overall monitoring accuracy of partial-penetration, full-penetration and burn-through etc. reached 98%. Liu et al. [24] used a deep-learning algorithm to predict the keyhole state in PAW, and built a perforation/penetration state prediction model for PAW. Taking the front molten pool images as the input of the model, the characteristic information of molten pool images was automatically extracted by CNN, and the recognition rate of perforation state on the prediction data sets was more than 90%. By using a BP (back propagation) neural network, Gu et al. [25] built a penetration state prediction model for K-TIG welding which took the geometric parameters of the molten pool and keyhole entrance as the input and the penetration state of back weld as the output. The result showed that the prediction accuracy of this model was more than 96%, which realized a good prediction of penetration state. Similarly, by obtaining the characteristic information of keyhole and molten pool, the researchers have built a prediction model between the characteristic parameters and the penetration [26].

In one-side welding with back-formation, the fusion hole is usually needed to ensure penetration. The mechanisms and behaviors of fusion hole are different from those of keyholes in other perforation processes. Guo et al. [2] built a low-cost visual detection system for TIG (tungsten inert gas) welding, observing the dynamic behaviors of the fusion hole in the front side. They studied the relationship between the shape parameters and the instability of the fusion hole, and built a mathematical analytical model of the characteristic parameters along with the longitudinal instability of the fusion hole. It was found that, when the welding current was 85 A, the fusion hole reached a quasisteady state after growing up, and the ratio of the fusion hole width to gap width fluctuated between 1.8 and 1.9. When the welding current reached 90 A, the ratio of the fusion hole width to gap width increased rapidly and fluctuated between 2.6 and 2.7 in five seconds after the start of welding, due to the accumulation of heat. The fusion hole became unstable and the weld was burnt through. Li et al. [27,28] observed the dynamic behaviors of fusion hole in 304 stainless steel with a thickness of 3 mm, and studied the influences of different welding parameters on the dynamic behaviors of fusion hole. It was found that, with the increase of welding current, the forming time of the fusion hole decreased and the length and width of the fusion hole increased. The ratio of the fusion hole length to width decreased slightly, due to the greater influence of welding current on the increase of fusion hole width. The above research was aimed at the welding of thin plates. Similarly, there are fusion holes in backing welding with reserved gap of the thick plate, but the images of the welding area obtained in thick plate welding are obviously different from those in thin plate welding. Most of the prior prediction models are based on the shape of the molten pool, keyhole and fusion hole to determine the penetration state of the weld. Because of the strong interference of arc radiation, wall reflection and other factors, it is difficult to obtain the morphological characteristics of the molten pool and fusion hole accurately all the time and the image processing process is complex.

**Figure 1:**
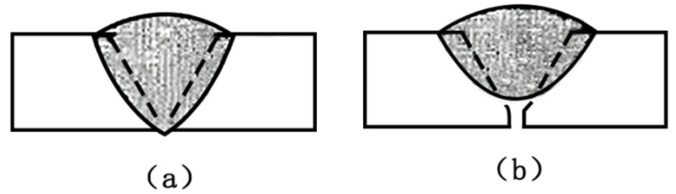
Two states of the weld: (**a**) penetration state of weld; (**b**) nonpenetration state of weld.

In the backing welding process of the thickness plate, it was found that the arc shape changes obviously along with the perforation of the fusion hole. The arc edge in the fusion hole image is clearer than the molten pool and fusion hole edge. In this paper, a passive visual detection method is used to collect the fusion hole images in TIG welding, and an image processing algorithm is designed by the MATLAB commercial software to obtain the arc characteristic parameters of fusion hole images. Taking the arc characteristic parameters as the model input, a prediction model for the penetration process of TIG welding is built. The different states of weld are shown in Figure 1. The perforation states of fusion hole which represents the penetration of weld are classified by SVM (support vector machine). This paper lays a foundation for penetration control of an automatic backing welding process for thick plate. 

## 2. Experimental Details

### 2.1. Visual Sensing System Design

**Figure 2:**
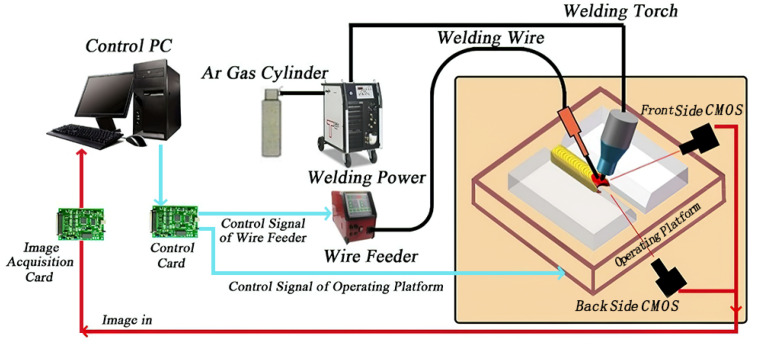
Tungsten inert gas (TIG) welding experimental platform.

The overall scheme of the TIG welding experimental platform is shown in Figure 2. The experimental platform was mainly composed of a TIG welding machine, a water-cooled welding torch, a wire feeder, two CMOS (complementary metal oxide semiconductor) cameras, a control unit, and a data acquisition card. The control unit was used to regulate the welding speed and the wire feed rate, as well as other welding parameters.

**Figure 3:**
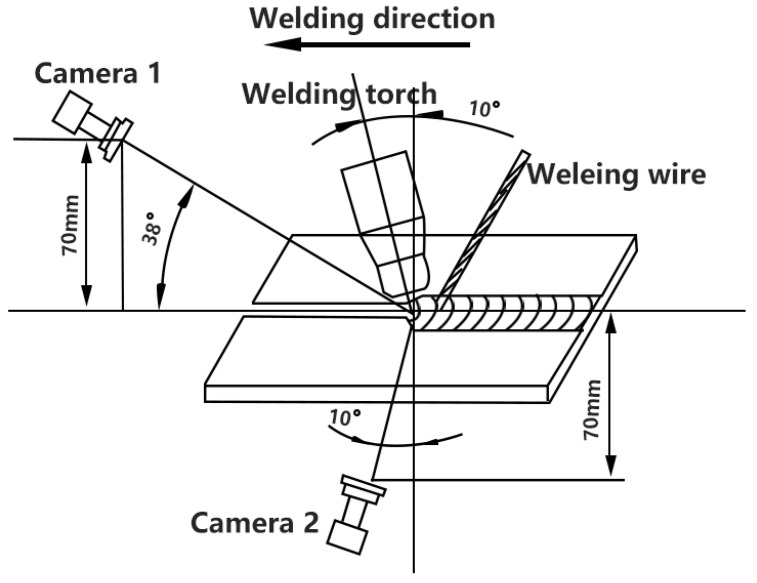
Camera position.

Camera 1 was fixed with the welding torch and the tungsten electrode is in the center view of camera 1. Camera 2 was fixed by a tripod. The relative position of the two cameras is shown in Figure 3. Camera 1 was equipped with a narrow-band filter with a central wavelength of 1064 nm, while camera 2 had a narrow-band filter with a central wavelength of 610 nm. The acquisition of front and back arc information was calibrated with the two central wavelength through testing. The two filters’ bandwidth was 30 nm and cutoff depth was OD4(optical density 4).

### 2.2. Experimental Method

**Figure 4:**
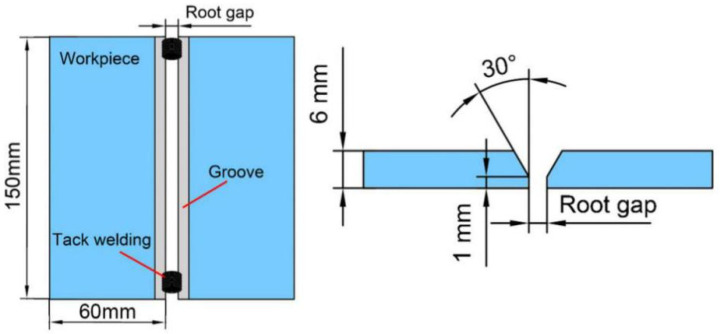
Size of the test plate.

The material used in this experiment was Q235 steel plate with the size shown in Figure 4. The welding wire was fed from the rear of the molten pool. The welding conditions were as follows: the tungsten electrode diameter was 3.0 mm, the shielding gas was argon (purity 99.99%), the gas flow rate was set to 15 L/min, the arc length was 3 mm, and the gap reserved was 2 mm. Tack welding was used at both ends of the test plate in advance to ensure that the reserved gap was constant. The welding parameters are shown in Table 1.

### 2.3. Visual Sensing System Design

In this paper, a pinhole model was selected to calibrate CMOS cameras without considering the error caused by lens distortion, which affects the accuracy little and could be neglected. A group of concentric circles with an adjacent distance of 2 mm was used as the calibration target. 

The *x*-axis was perpendicular to the direction of welding, and the *y*-axis was parallel to the direction of welding. *K_x_* and K*_y_* represent the calibration coefficients of camera 1 on *x*-axis and *y*-axis respectively, and the calibration results were:

(1) $$K_{x}=0.01482$$

(2) $$K_{y}=0.02352$$

## 3. Test Data Acquisition

### 3.1. Label of Fusion Hole State 

**Figure 5:**
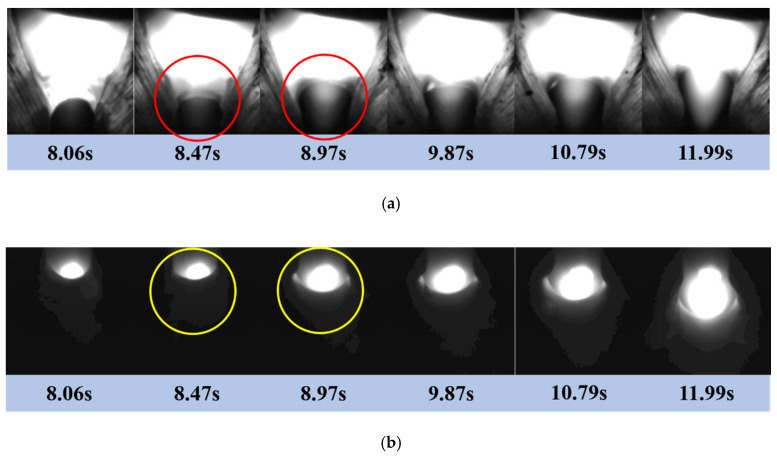
Typical perforation process of fusion hole: (**a**) The front images of fusion hole; (**b**)The backside images of fusion hole.

With the welding speed at 100 mm/min, the welding current at 140 A and the wire feed rate at 95 cm/min, a typical perforation process of fusion hole was obtained and is shown in Figure 5.

After a striking arc in the area of tack welding, the base metal and filler metal in the area were melted under the action of arc heat input. As the welding process continued, the liquid metal in the tack welding area entered the reserved gap, the thickness of the molten pool becomes thinner, and the front end of the molten pool became crescent shaped. The arc acted directly on the front surface of the molten pool, making the temperature of this area higher than that of the surrounding area, and the surface tension in the front end of the molten pool decreased. Under the coupling effect of arc pressure, arc heat and surface tension of molten pool, the crescent shaped liquid in the front end of the molten pool was forced to move backward, forming a semiopen hole—the fusion hole. At 8.97 s, the fusion hole was perforated, as shown in the circle in Figure 5b.

The arc area in the circle shown in Figure 5a changed obviously before and after fusion hole perforation. With the increase of fusion hole size, the arc became more concentrated and the area which the arc goes through increased. Therefore, the arc area inside the fusion hole could reflect the characteristic information of fusion hole perforation.

In this paper, the images of the whole welding process are labeled artificially. The non perforation image is labeled as false, which is a negative sample, and the value is 0; the perforation image is labeled as true, which is a positive sample, and the value is 1.

### 3.2. Image Processing

**Figure 6:**
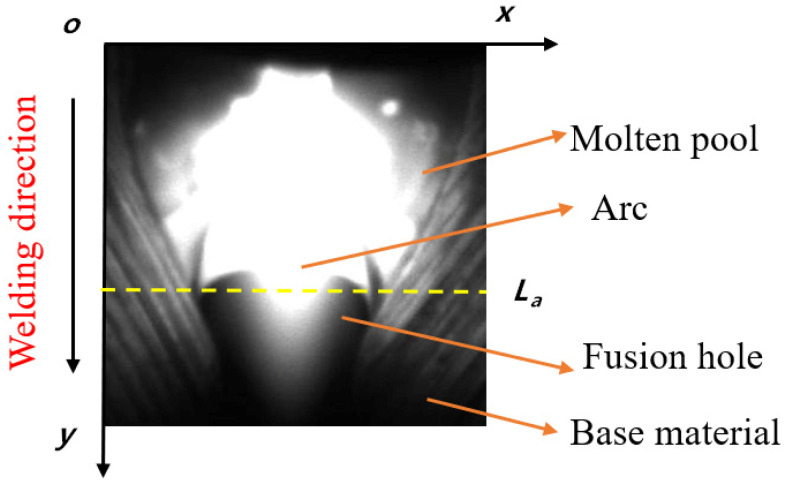
Image of fusion hole in the front side.

In order to ensure that the weld was penetrated, the fusion hole had to be throughout the work-piece ideally. Figure 6 shows the front image of fusion hole taken by camera 1.

**Figure 7:**
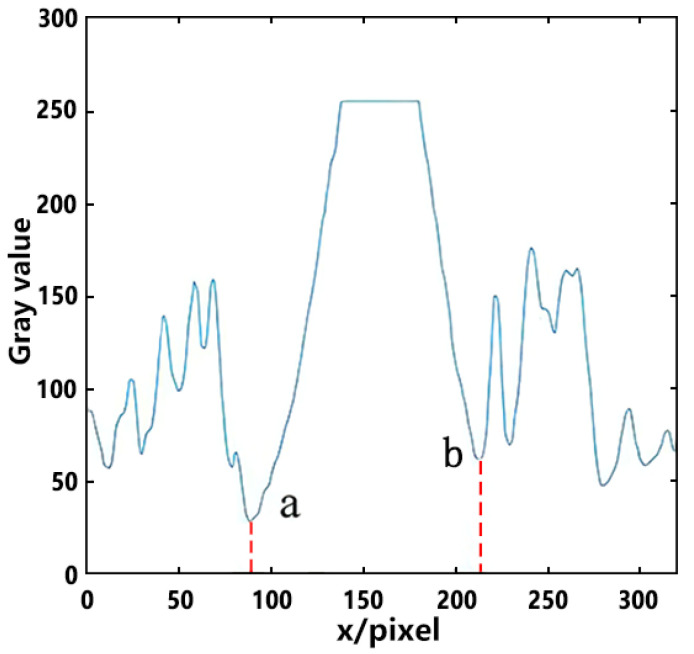
Gray scale distribution in La line.

In the welding process, the arc light which wasn’t reflected to the visual sensor passed through the area of fusion hole and reserved gap, then entered the back side. The arc itself emitted light, so most of the area of the fusion hole and reserved gap was black or gray, and the arc area was bright. The gray scale of line La is extracted in Figure 6 and displayed in Figure 7. The area with a high value of gray scale is the arc center; due to the radiation effect of arc, the further away from the arc center the area is, the lower the gray scale value is. The first minimum points a and b appear on both sides of the center line, and the area between a and b is taken as the arc area.

**Figure 8:**
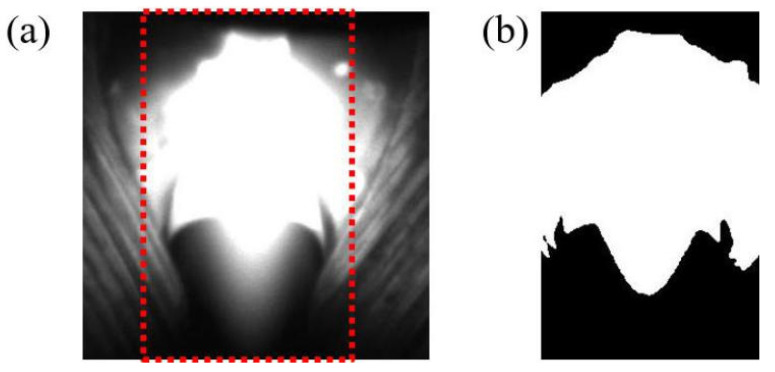
Arc contour obtained by OTSU method: (**a**) region of interest; (**b**) threshold segmentation.

A fixed size window was used to process the images of the arc area. Only part of the arc area is contained in the window shown in Figure 8a, which is the region of interest. The region of interest in red box is intercepted with the size of 100 × 320 pixel, which contains the characteristic information of the arc that must be extracted. The OTSU [29] method was used for threshold segmentation. The image processed by the OTSU method is shown in Figure 8b, and the contour of the front end of the arc is clear.

**Figure 9:**
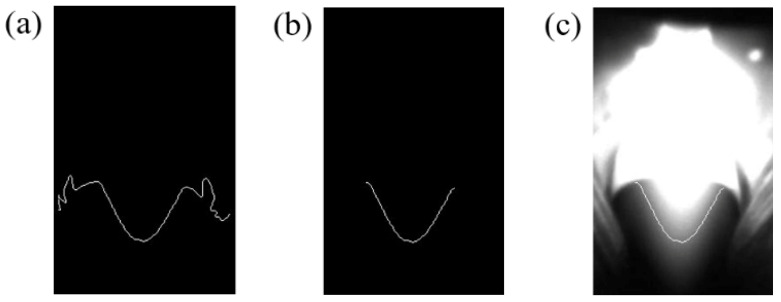
Processing results with fusion hole: (**a**) canny operator; (**b**) extra arc edge removed; (**c**) edge in image.

**Figure 10:**
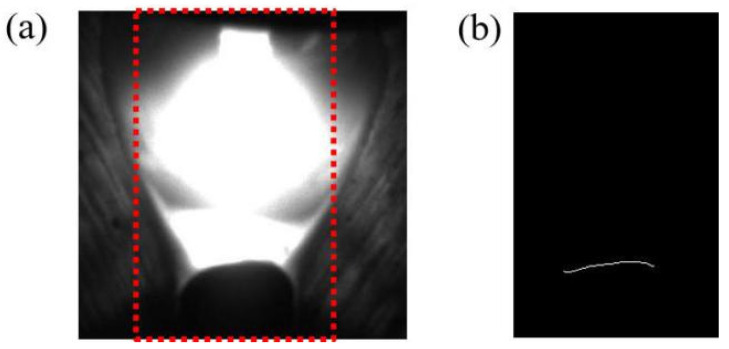
Processing result without fusion hole: (**a**) canny operator; (**b**) extra arc edge remove.

When extracting the edge of arc area, the position relationship between the arc and the fusion hole must be considered. Part of the arc travels through the fusion hole when the fusion hole forms, and the front end of the arc is parabolic. The steps of the edge detection algorithm were as follows: (1) a canny [30] edge detection operator was used to detect the edge, and the design algorithm only extracted the lower edge of the arc contour, as shown in Figure 9a; (2) a method based on minimum value was used to remove the extra arc edge and the edge was in the shape of a “parabola”, as shown in Figure 9b. It can be seen from Figure 9c that the edge was well matched. Figure 10 shows the result of image processing without the fusion hole and the edge was almost straight. It can be seen from Figure 9 and Figure 10 that there was a big difference between the arc edges with and without fusion holes.

### 3.3. Characteristic Parameters Extracting

**Figure 11:**
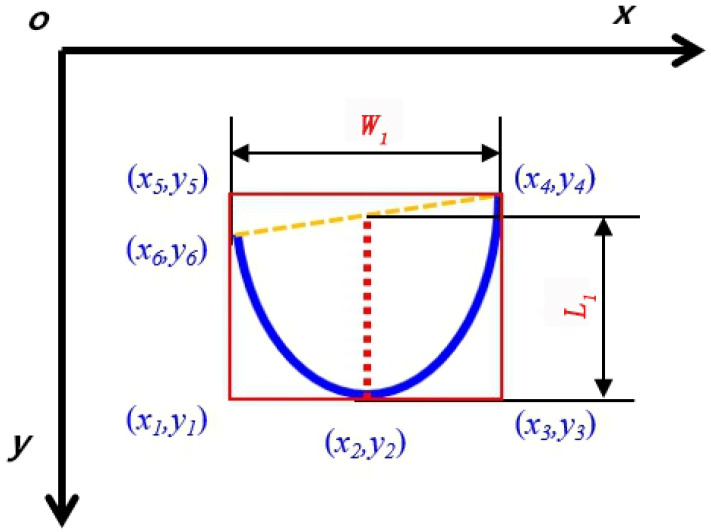
Characteristic image of the arc front end.

The typical morphological characteristics of the front end of the arc were defined when the fusion hole exists, as shown in Figure 11. The defined characteristics included the front end width of arc (*W*_1_), the front end length of arc (*L_1_*) and the width/length ratio of edge curve (*W/L*). The width/length ratio of the edge curve was equal to the width/length ratio of the minimum circumscribed rectangle of the edge curve. 

The image was searched line by line from *y =* 1 to *y = n* along the *x* direction. The coordinates of the outermost points on the left and right sides of the curve are (*x*_6_*,y*_6_) and (*x*_4_*,y*_4_) respectively. The calculation formula of the arc front end width (*W_1_*) is as follows:

(3) $$W_{1}=K_{x}(x_{6}-x_{4}),$$

Then the image was searched line by line along the y direction and the lowest point (*x_2_,y_2_)* of edge curve was acquired. The calculation formula of arc front end length (*L_1_*) is as follows:

(4) $$L_{1}=K_{y}[(y_{6}-y_{2})+(y_{4}-y_{2})]/2,$$

The width/length ratio (*W/L*):

(5) $$W/L=K_{x}(x_{5}-x_{4})/[K_{y}(y_{5}-y_{1})],$$

The relationships between the points in Figure 11 are as follows: point (*x*_5_*,y*_5_) and point (*x*_4_*,y*_4_) are on the identical line, which is parallel to *x*-axis; point (*x*_1_*,y*_1_), point (*x*_2_*,y*_2_) and point (*x*_3_*,y*_3_) are on the identical line, which is parallel to the *x*-axis; point (*x*_1_*,y*_1_), point (*x*_6_*,y*_6_) and point (*x*_5_*,y*_5_) are on the identical line, which is parallel to *y*-axis; point (*x*_3_*,y*_3_) and point (*x*_4_*,y*_4_) are on the identical line, which is parallel to the *y*-axis.

Since the curve of the arc front end with fusion hole is approximately parabolic, the parabola approximation algorithm was used to fit the curve, and the pixel coordinates are recorded as follows:

(6) $$\left\{\left(x_{j},y_{j}\right)\right\}\left\{j=0,1,2\ldots n\right\},$$

Fit function *t(x_j_)*:

(7) $$t(x_{j})=ax_{j}^{2}+bx_{j}+c,$$

The coefficients *a*, *b* and *c* are the fitting coefficients.

The sum of square errors is:

(8) $$E^{2}=\left({\sum \left[t\left(x_{j}\right)-y_{j}\right]}^{2}\right),$$

In the parabolic curve model, the fitting coefficient can represent the characteristics of the parabola. In this paper, the coefficients *(a, b, c)* of fitting function are taken as the characteristic parameters.

**Figure 12:**
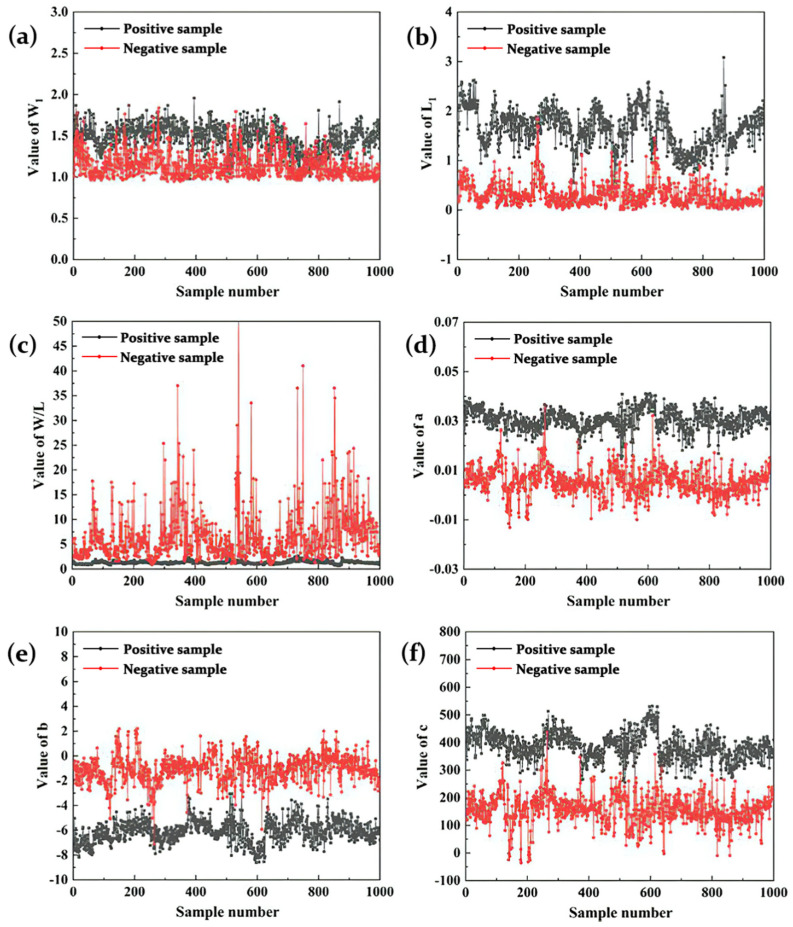
Experimental data of training samples: (**a**) *W*_1_; (**b**) *L*_1_; (**c**) *W*/*L*; (**d**) *a*; (**e**) *b*; (**f**) *c*.

About 250 different fusion hole images were extracted from each experiment. In all the image samples, 2000 images were selected as training samples and 300 images were selected at welding current of 130A as the prediction samples. There was no overlap between training samples and the prediction samples. Figure 12 shows the characteristic parameters of training samples. It can be found that the positive and the negative samples can be clearly distinguished by the characteristic parameters.

## 4. Results and Discussion

### 4.1. Fusion Hole Classification Model

In this paper, the SVM classification method integrating with the image processing technology was used to predict the perforation process of fusion hole. The basic idea of this model was to map the sample data to high-dimensional space through nonlinear mapping, and transform it into a linear separable problem.

The modeling processes of SVM prediction model were as follows:

(1) Based on the training data obtained, the cross validation and the grid-search method were used to find the optimal kernel function *g* and the penalty function *c*.
(2) The SVM was trained with the training data to obtain the support vector and the model structure was determined.
(3) The model was used to predict the prediction samples.

**Figure 13:**
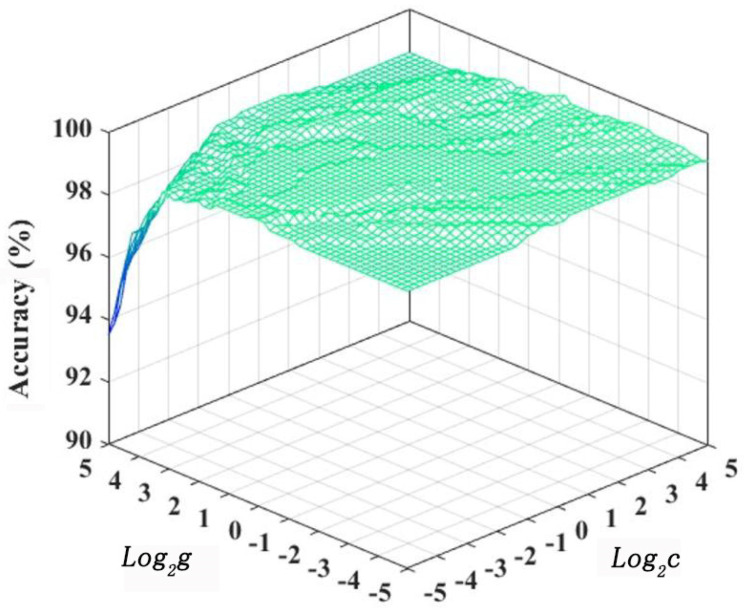
Grid-searching result of training parameters.

Grid-searching results of training parameters are shown in Figure 13. The highest accuracy was 99%, and most of the accuracy was higher than 90%, proving that there exists arc characteristic parameters which can represent the characteristics of fusion hole perforation. When the accuracy of the test set was highest, the corresponding *Log_2_c* and *Log_2_g* were taken to obtain the penalty function *c* and the kernel function *g*. In this paper, *Log_2_c =* 0 and *Log_2_g =* 0 were chosen, and *c* and *g* were both equal to 1.

### 4.2. Prediction of Fusion Hole Formation Based on SVM

In the field of machine learning, parameters such as accuracy (Acc), precision (P), recall and F1-measure are often used to measure the performance of classifiers. The specific formulas are based on the parameters in Table 2. The calculation method of each formula is shown in Table 3.

The weld was not always penetrated when the fusion hole did not perforate. When the model predicts nonperforation as perforation, the system will misjudge that the weld has penetrated at this time, which has a vital impact on the weld forming quality. Therefore, the number of negative samples misjudged by the model as positive ones must be strictly controlled, and even equal 0. When the model predicted the perforation samples as nonperforation ones, the weld is penetrated, which had little effect on the model. If there was little difference in accuracy, the higher the P was, the better the predict result was.

**Figure 14:**
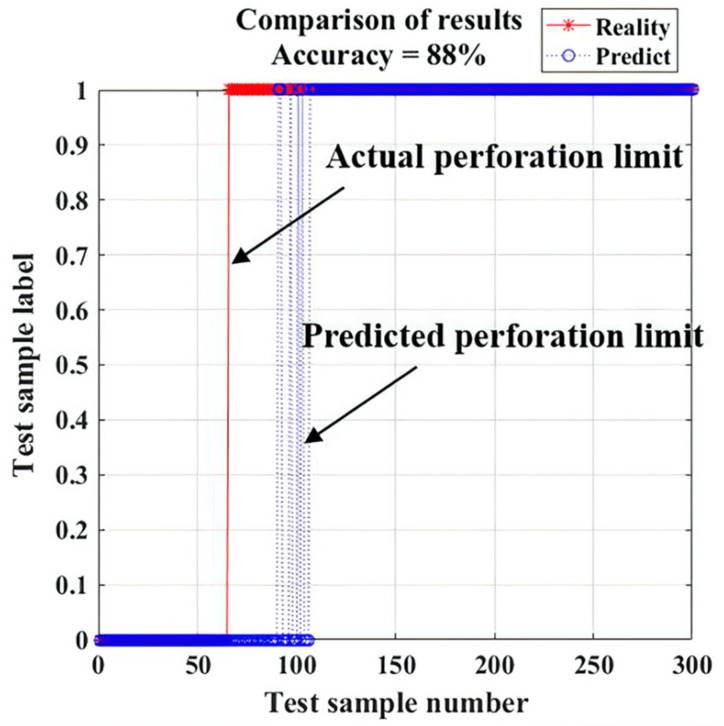
Experimental result.

The prediction results are shown in Figure 14 and Table 4. According to the data, the critical point from nonperforation to perforation was defined as the perforation limit.

The experimental result shows that the Acc and the F1-Measure were slightly above or below 90% and the precision was 100%, which means that the prediction model had a strong detection ability for positive samples, and it was difficult to predict the images of nonperforation as the ones of perforation. For the images of perforation, the true accuracy was 84.68%, and there were no misidentified samples after the predicted perforation limit, which proves that the characteristic parameters of arc can obviously characterize the perforation and can effectively be selected to predict perforation.

**Figure 15:**
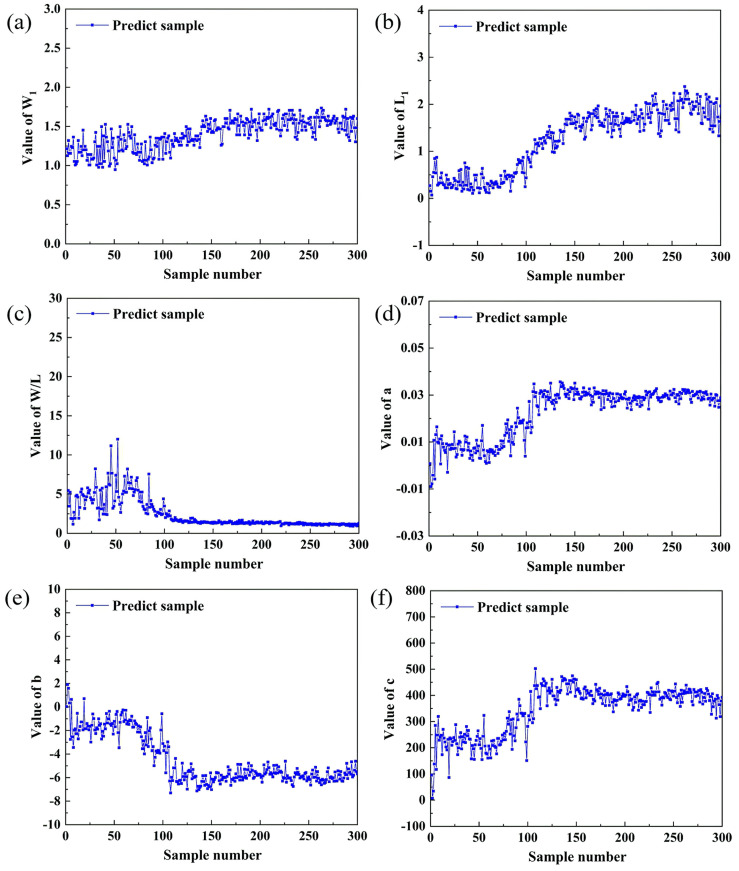
Experimental data of prediction samples: (**a**) *W*_1_; (**b**) *L*_1_; (**c**) *W*/*L*; (**d**) *a*; (**e**) *b*; (**f**) *c*.

**Figure 16:**
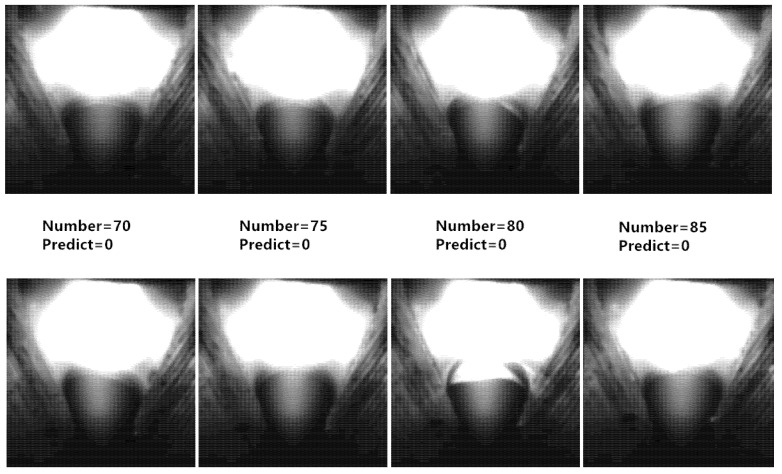
Misidentified images.

The experimental data of prediction samples are shown in Figure 15. In the nonperforation stage, the characteristic parameters of the arc tended to be steady. In the period between actual perforation and predicted perforation limit, *W_1_*, *L_1_*, *a* and *c* increased constantly, while *W/L* and *b* decreased. After the predicted perforation limit, the characteristic parameters of the arc tended to be steady again. The characteristic parameters of the arc in the nonperforation stage were different from those in the stage after predicted perforation limit, and the two sets of parameters were stratified obviously, which proves that the characteristic parameters of the arc can obviously characterize the perforation again. The misjudged samples were concentrated in the initial stage of perforation, and Figure 16 shows the misidentified images. As the fusion hole was just perforated, the arc inside the arc area was weak. Although all the characteristic parameters of the arc changed, there was no obvious difference of the parameters’ values in this stage compared with the nonperforation stage. With the increase of fusion hole size, the arc characteristic parameters all changed obviously, and then tended to be stable.

After stable perforation, the perforation state could be easily identified. The predicted perforation limit time was 0.8 s later than the actual time, and there were about 40 images in this period. When the fusion hole perforation state was given by the model, the fusion hole already had a certain size, which ensured the weld was penetrated.

## 5. Conclusions

(1) A visual detection system of a fusion hole in TIG backing welding was built, and clear images of the front and the back fusion hole were obtained.
(2) With the increase of fusion hole size, the arc gradually passed through the fusion hole, and there were obvious differences between the arc before and after fusion hole perforation. The arc characteristics of front fusion hole images could be used as the basis for judging the state of the fusion hole.
(3) The image processing method was designed and the arc characteristic parameters were obtained.
(4) Taking arc characteristic parameters as the model input, penalty function and kernel function parameters were obtained by cross validation and grid-search method. A prediction model of fusion hole perforation in TIG welding based on SVM was constructed. The results show that the classification accuracy of the model reached 88%, and the time of prediction perforation limit was slightly later than the actual one, which was conducive with ensuring the weld penetration.

## Figures and Tables

**Table 1:** Welding parameters.

Test Case No.	Welding Current (A)	Welding Speed (mm/min)	Wire Feeding Speed (mm/min)
CD1–2–3	120–130–140	100	95
CS1–2-3	120	100	85–95–105
CJ1	120	100	95
BSD-1	120	100–120	85
BSD-2	120	100–80	85
BSS-1	120	100	95–135–95

**Table 2:** Statistical parameters of prediction result and actual result.

Actual Result	Prediction Result	
	Positive	Negative
Positive	True Positive (TP)	False Negative (FN)
Negative	False Positive (FP)	True Negative (TN)

**Table 3:** Calculation method of formula

Category	Accuracy	Precision	Recall	F_1_-Measure
Formula	$\mathrm{Acc}=\frac{\mathrm{TP}+\mathrm{TN}}{\mathrm{total}}$	$P=\frac{\mathrm{TP}}{\mathrm{TP}+\mathrm{FP}}$	$R=\frac{\mathrm{TP}}{\mathrm{TP}+\mathrm{FN}}$	$F_{1}=\frac{2\times P\times R}{P+R}$

**Table 4:** Experimental result.

Category	Accuracy	Precision	Recall	F_1_-Measure
Value-SVM	88.00%	100.00%	84.68%	91.70%
